# Supplementary material for: Genomic surveillance of COVID-19 cases in Beijing
Source: Nat Commun. 2020 Oct 30;11:5503. doi: 10.1038/s41467-020-19345-0 (PMC7603498; doi:10.1038/s41467-020-19345-0)
Supplement: Supplementary file 1 — Supplementary Information [file 41467_2020_19345_MOESM1_ESM.pdf]

Supplementary Figure 1. The geographic sources and the occurrence time of COVID-19 patients in Beijing. (A) The distribution of cases from different countries and regions. The data were from the reports of Beijing Municipal Health Commission (http://wjw.beijing.gov.cn/). All of the maps in this study were derived from the standard world map (No. GS[2016]2968) from the Ministry of Natural Resources of the People's Republic of China (http://bzdt.ch.mnr.gov.cn/browse.html?picId=%224o28b0625501ad13015501ad2bfc0116%22), which is freely provided to the public for publication. (B) The number of COVID-19 cases in Beijing till April 17, 2020. The brown histograms represent the daily case numbers and the orange line was the curve of accumulative case number. (C) The numbers of cases from different countries and regions.

A

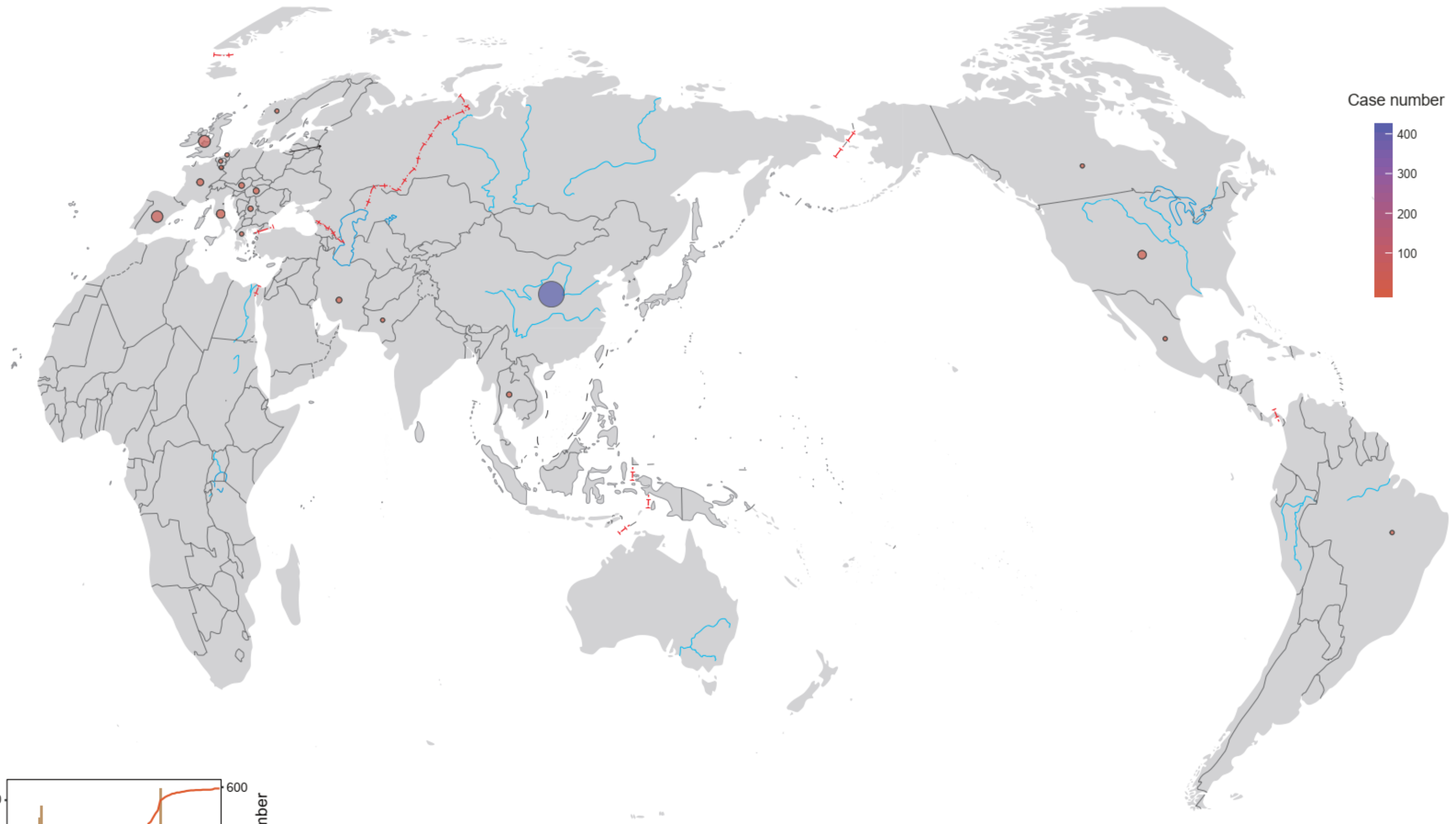

B

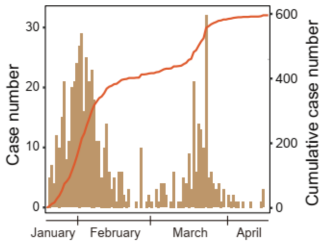

C

Source of COVID-19 cases

	China		Asia			Europe												North America		South America	
Source	Local	Import(total)	Iran	Tailand	Pakistan	UK	Spain	Italy	France	Serbia	Hungary	Austria	Netherlands	Belgium	Luxembourg	Norway	Greece	USA	Canada	Brazil	Mexico
Mar 31	416	164	3	2	1	53	46	18	7	2	4	3	1	1	1	1	1	17	1	1	1

Supplementary Figure 2. The genomic position and associated genes of the 17 high frequently SNPs. The arrow chart on the top represents the protein coding genes of SARS-CoV-2.

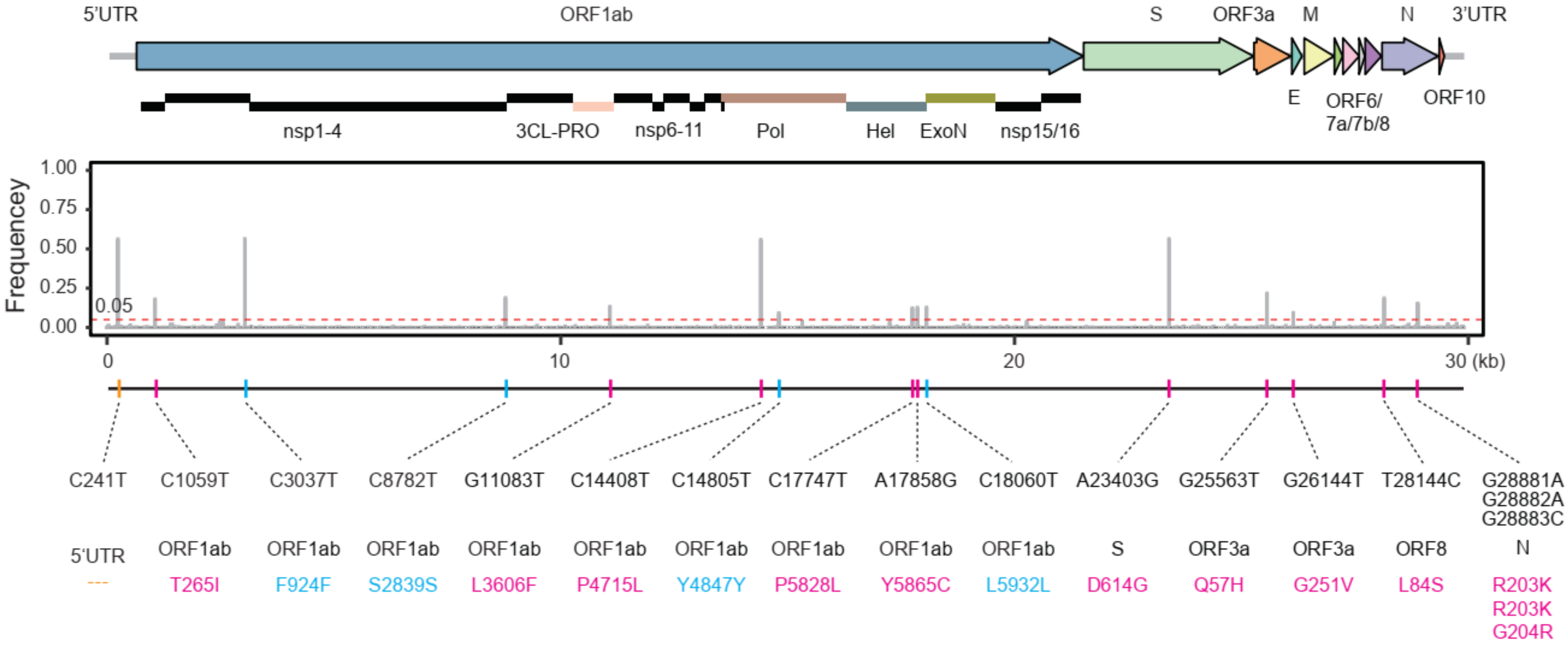

Supplementary Figure 3. The phylogenetic tree of the public genomes and those we sequenced enrolled in this study. The tips of the tree are colored by the clusters we defined. The lineage nomenclature by Rambaut et al are marked by two rings outside. The lineages are in different colors. Source data are provided as a Source Data file.

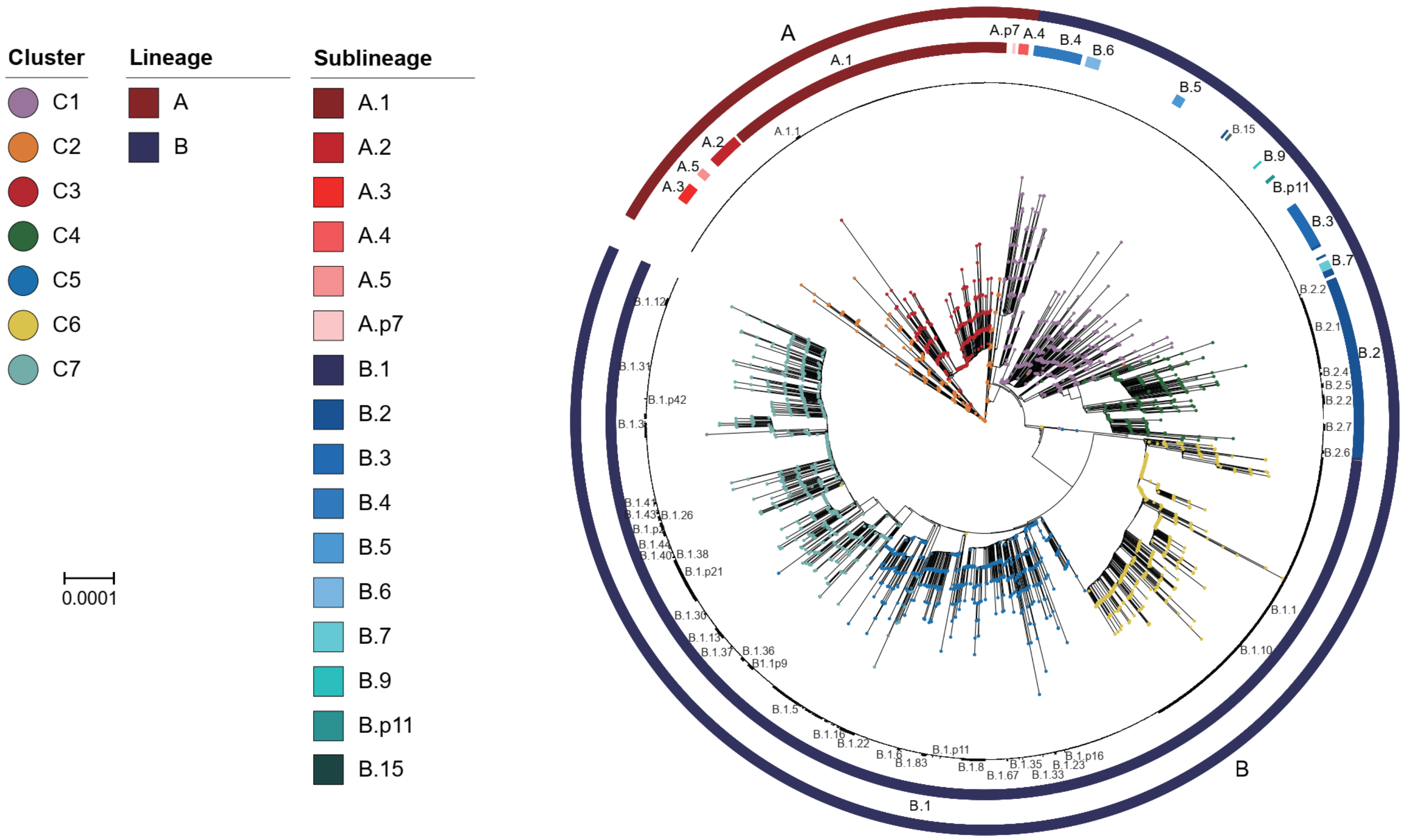

Supplementary Figure 4. Lineage determination of the viral genomes sequenced in this study. (A) The sublineages of genomes we sequenced in each cluster. (B) The numbers of virus of different lineages emerged in the three groups during the outbreak. Source data are provided as a Source Data file.

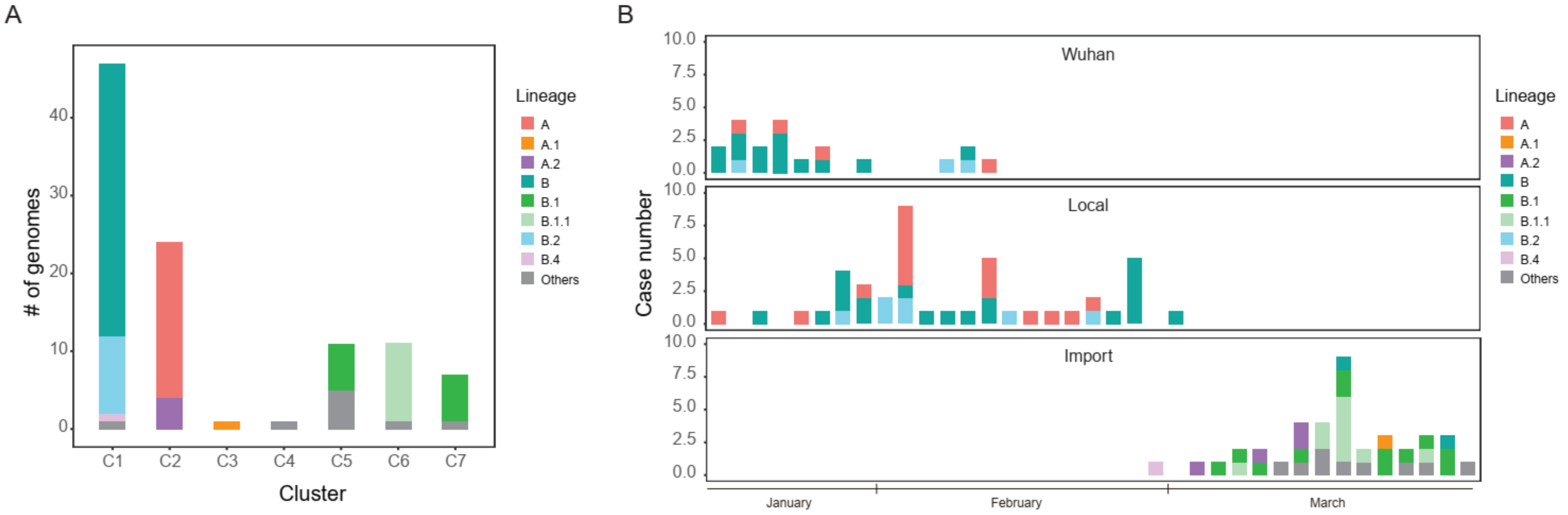

Supplementary Figure 5. The emerging times and genome numbers of the seven clusters. Source data are provided as a Source Data file.

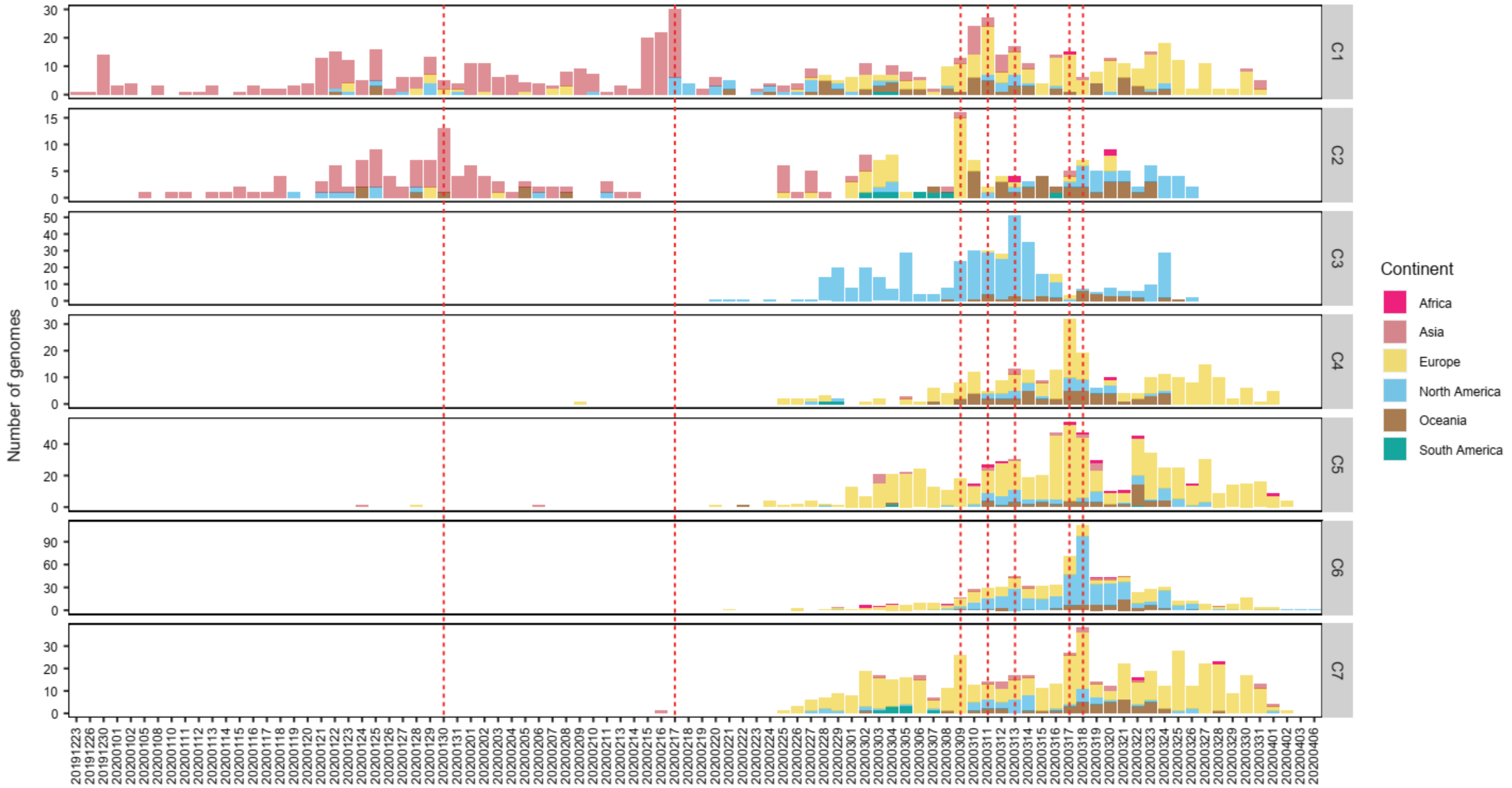

Supplementary Figure 6. The distribution of the seven clusters in different continents. Source data are provided as a Source Data file.

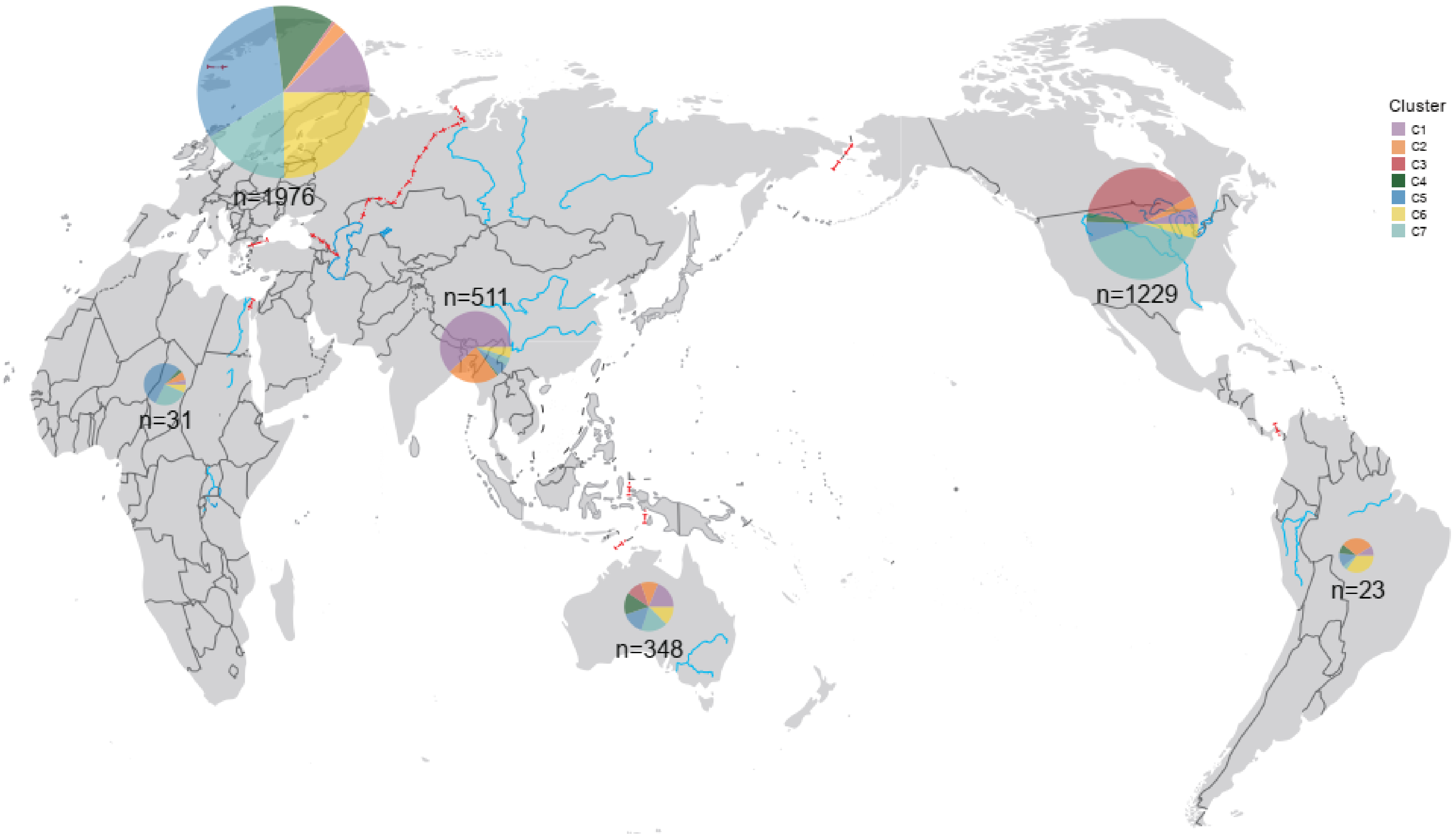

Supplementary Figure 7. The differences of numbers of SNPs and iSNVs along with the age, gender, disease level and sources of the cases in Beijing. The two-sided Wilcoxon tests (two groups) or Kruskal-Wallis (more than two groups) were performed to compare the difference. Box plots indicate median (middle line), the first and third quartiles (box), the first quartile minus 1.5-fold the interquartile range and the third quartile plus 1.5-fold the interquartile range.

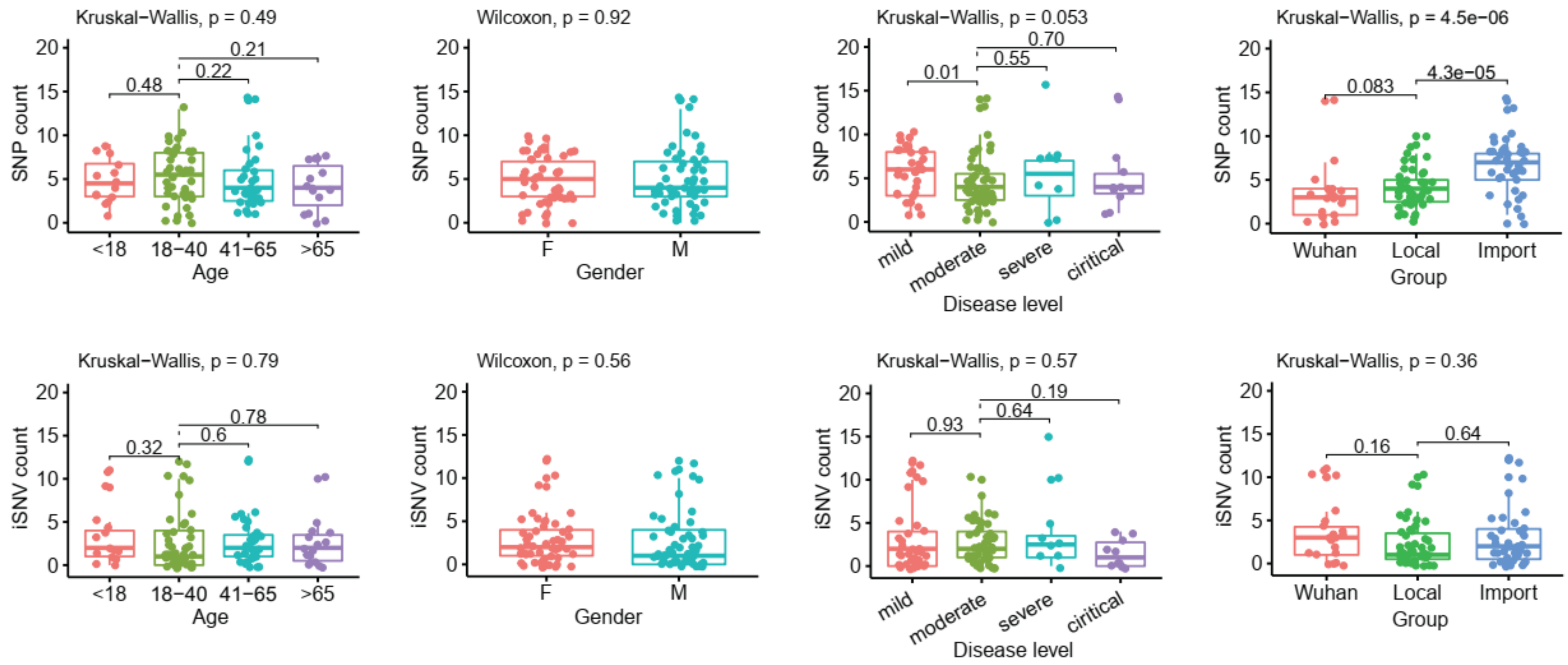

Supplementary Table 1. COVID-19 samples sequenced in this study.

Sample	Group	Sex	Disease level	Sample type	Total reads	Genome% (5X)	Average depth
S001	Wuhan	F	ciritical	pharyngeal swabs	13884580	0.903822	650.009
S002	Local	M	ciritical	pharyngeal swabs	12387588	0.997927	17778.3
S003	Wuhan	M	ciritical	pharyngeal swabs	13025918	0.915895	2236.05
S004	Local	M	ciritical	pharyngeal swabs	16384556	0.989733	4480.94
S005	Wuhan	F	moderate	sputum	207709272	0.999967	88671.8
S006	Wuhan	M	moderate	sputum	356396564	0.999933	93085.1
S007	Local	M	ciritical	pharyngeal swabs	541628130	1	93803.7
S008	Import	M	mild	pharyngeal swabs	201614222	0.99582	74201.3
S009	Import	F	mild	sputum	116156350	0.999431	85212
S010	Local	M	moderate	sputum	146373482	0.999565	92525.9
S011	Local	F	moderate	sputum	66509586	0.98465	276.117
S012	Local	F	severe	pharyngeal swabs	43022752	0.999365	74459.2
S013	Wuhan	M	ciritical	pharyngeal swabs	29918078	0.99592	47678.5
S014	Local	F	ciritical	pharyngeal swabs	63305808	0.998963	84928.4
S015	Local	F	severe	pharyngeal swabs	28502096	0.980437	1358.45
S016	Wuhan	M	moderate	pharyngeal swabs	28763804	0.986189	2026.11
S017	Wuhan	M	moderate	pharyngeal swabs	48033536	0.999164	11247.8
S018	Wuhan	M	moderate	pharyngeal swabs	32374174	0.996121	4607.07
S019	Wuhan	F	mild	pharyngeal swabs	33545522	0.900244	669.763
S020	Wuhan	F	moderate	pharyngeal swabs	41228324	0.99679	933.399
S021	Import	M	mild	pharyngeal swabs	12177692	0.997325	18520.3
S022	Import	F	moderate	pharyngeal swabs	6105636	0.996622	6216.07
S023	Import	F	moderate	pharyngeal swabs	6556106	0.999298	4669.93
S024	Import	F	moderate	pharyngeal swabs	45398712	0.999699	72139.5
S025	Import	M	mild	pharyngeal swabs	103807862	0.999465	90222.6
S026	Import	F	mild	pharyngeal swabs	5072904	0.997392	2395.14
S027	Import	F	mild	pharyngeal swabs	26651568	0.999097	46588.5
S028	Import	F	mild	faeces	119250340	0.999131	92137.3
S029	Import	F	mild	faeces	13876992	0.998662	18676.7
S030	Import	F	moderate	sputum	6685592	0.997425	1860.61
S031	Import	M	mild	sputum	6482172	0.887001	266.996
S032	Import	M	moderate	sputum	5970510	0.997994	3691.24
S033	Import	M	moderate	sputum	8697652	0.999097	8769.17
S034	Import	F	moderate	sputum	25258898	0.999264	39615.1
S035	Import	F	mild	sputum	141070280	0.999599	92417.4
S036	Import	F	severe	sputum	9016134	0.998194	3406.15
S037	Local	M	moderate	pharyngeal swabs	7900584	0.996121	1182.58
S038	Local	F	ciritical	pharyngeal swabs	8211094	0.998696	6934.27
S039	Local	F	moderate	pharyngeal swabs	53204030	0.998896	64571.4
S040	Local	M	severe	pharyngeal swabs	87417894	0.997826	75361.9
S041	Local	M	ciritical	pharyngeal swabs	3872946	0.983781	6107.21
S042	Local	M	moderate	faeces	4647760	0.972712	812.386
S043	Local	M	moderate	sputum	1267440	0.922349	1633.24
S044	Local	M	moderate	sputum	75641774	0.999331	82143.1
S045	Local	F	moderate	sputum	40132820	0.998094	32484.9

S046	Wuhan	M	moderate	sputum	21566432	0.997325	5067.33
S047	Local	F	moderate	sputum	18074214	0.999097	39345.8
S048	Import	F	moderate	sputum	16104054	0.995987	10682.1
S049	Import	M	mild	sputum	9204622	0.999298	16902.9
S050	Import	M	ciritical	pharyngeal swabs	39599530	0.917868	30350.9
S051	Import	F	mild	sputum	39323718	0.998294	28307.8
S052	Import	M	mild	pharyngeal swabs	13402228	0.949671	4380.52
S053	Import	F	moderate	sputum	3782978	0.894459	126.608
S054	Import	F	moderate	sputum	13857778	0.989265	5128.14
S055	Import	F	mild	sputum	19135698	0.992208	6532.67
S056	Import	F	mild	sputum	130819780	0.999599	89571.9
S057	Import	F	mild	sputum	15273002	0.916263	12742.4
S058	Import	M	mild	pharyngeal swabs	16728222	0.921245	2904.76
S059	Wuhan	M	moderate	sputum	14792758	0.946962	3850.46
S060	Wuhan	M	moderate	sputum	10026796	0.999264	16009.2
S061	Local	F	moderate	sputum	23105612	0.99883	39581.1
S062	Local	F	moderate	sputum	34368266	0.998696	38022.6
S063	Local	M	moderate	sputum	54788752	0.999331	79724.8
S064	Wuhan	M	severe	pharyngeal swabs	35601376	0.997425	3947
S065	Wuhan	F	severe	pharyngeal swabs	23099268	0.993947	1536.95
S066	Import	F	mild	sputum	35048174	0.998194	48357.6
S067	Local	F	mild	faeces	39411066	0.981774	7692.83
S068	Local	M	moderate	faeces	36100522	0.997994	58174.4
S069	Local	F	moderate	faeces	23008060	0.994148	3893.16
S070	Local	M	moderate	faeces	34833814	0.997458	28627.3
S071	Local	M	moderate	sputum	11775478	0.998361	11184.8
S072	Local	F	moderate	sputum	28157176	0.869846	6038.45
S073	Local	M	moderate	sputum	25806944	0.972511	4557.25
S074	Local	M	moderate	sputum	40811206	0.962847	17941.9
S075	Wuhan	M	mild	faeces	15901590	0.999833	27991.4
S076	Local	M	mild	pharyngeal swabs	139565182	0.880581	20234.3
S077	Local	M	mild	faeces	245791438	0.902017	22759.9
S078	Import	M	mild	faeces	69065232	0.998228	17444.6
S079	Local	F	moderate	pharyngeal swabs	252038586	0.999967	87253
S080	Local	M	moderate	sputum	96238068	0.997826	66248.2
S081	Local	F	moderate	pharyngeal swabs	108884390	0.993345	47767.9
S082	Local	F	mild	faeces	374145632	0.998294	91808.5
S083	Local	F	moderate	pharyngeal swabs	190930830	0.997592	85840.1
S084	Local	F	mild	pharyngeal swabs	72845794	0.996656	25390.8
S085	Local	M	moderate	sputum	143745818	0.871719	30824.6
S086	Import	M	moderate	faeces	95943060	0.991004	4008.84
S087	Local	F	mild	faeces	185858560	0.998395	84976.2
S088	Import	M	moderate	faeces	72877922	0.966023	33230.4
S089	Local	F	mild	faeces	101412998	0.998529	56715
S090	Wuhan	F	moderate	pharyngeal swabs	127839752	0.999632	81086.4
S091	Local	M	moderate	sputum	90255466	0.998529	78622.5
S092	Wuhan	M	moderate	sputum	151281934	0.983714	30876.8
S093	Import	F	mild	sputum	133758300	0.906063	29699.1

S094	Local	M	moderate	faeces	113568406	0.990235	55446.3
S095	Wuhan	M	severe	pharyngeal swabs	142263848	0.933619	20512.5
S096	Import	M	mild	faeces	28131570	0.997392	16910.6
S097	Local	M	severe	sputum	58148400	0.940106	32687.6
S098	Wuhan	F	moderate	pharyngeal swabs	432164526	0.999699	89747.5
S099	Import	M	moderate	sputum	113258714	0.995084	37837.5
S100	Import	M	mild	sputum	138572392	0.999599	62594.3
S101	Import	F	mild	sputum	232942870	0.999933	85268.6
S102	Import	F	mild	sputum	93282332	0.999197	71012.1

Supplementary Table 2. The seventeen high frequency SNPs for the identification of seven viral clusters.

Position*	Gene	Ref. allele	Ref. codon	Ref AA	Position in protein	Alt allele	Alt codon	Alt AA	Mutation type
241	orf1ab	C	-	-	-	T	-	-	5'UTR
1,059	orf1ab	C	ACC	T	265	T	ATC	I	missense
3,037	orf1ab	C	TTC	F	924	T	TTT	F	synonymous
8,782	orf1ab	C	AGC	S	2839	T	AGT	S	synonymous
11,083	orf1ab	G	TTG	L	3606	T	TTT	F	missense
14,408	orf1ab	C	CCT	P	4715	T	CTT	L	missense
14,805	orf1ab	C	TAC	Y	4847	T	TAT	Y	synonymous
17,747	orf1ab	C	CCT	P	5828	T	CTT	L	missense
17,858	orf1ab	A	TAT	Y	5865	G	TGT	C	missense
18,060	orf1ab	C	CTC	L	5932	T	CTT	L	synonymous
23,403	S	A	GAT	D	614	G	GGT	G	missense
25,563	ORF3a	G	CAG	Q	57	T	CAT	H	missense
26,144	ORF3a	G	GGT	G	251	T	GTT	V	missense
28,144	ORF8	T	TTA	L	84	C	TCA	S	missense
28,881	N	G	AGG	R	203	A	AAG	K	missense
28,882	N	G	AGG	R	203	A	AGA	R	synonymous
28,883	N	G	GGA	G	204	C	CGA	R	missense

*According to the reference genome of Wuhan-Hu-1 (accession: NC_045512.2).

Supplementary Table 3. Ka/Ks based on SNPs we obtained in this study. The two-sided Fisher exact tests were performed to test the probabilities.

Gene	Method	Ka	Ks	Ka/Ks	P-Value (Fisher)
orf1ab	MA	0.000100946	0.000222161	0.454382	3.96E-10
orf1a	MA	0.000106728	0.000380105	0.280786	1.18E-17
S	MA	0.000408264	0.000146713	2.78273	0.0102363
ORF3a	MA	0.00153618	0.000425404	3.6111	0.0455809
E	MA	0.00552886	0.000110577	50	0
M	MA	0.00116379	0.00253081	0.459848	0.293135
ORF6	MA	0.00530816	0.00658324	0.806314	0.450222
ORF7a	MA	0.00372216	7.44E-05	50	0
ORF7b	MA	0.0100114	0.000206418	48.5005	0.352049
ORF8	MA	0.00485682	9.71E-05	50	3.64E-06
N	MA	0.00101921	0.00307866	0.331058	2.31E-05

Supplementary Table 4. The differences of numbers of SNPs and iSNVs along with the age, gender, disease level and sources of the cases in Beijing. The two-sided Wilcoxon tests were performed to compare the difference.

Characteristics	Patients No. (%)	SNP count median (qu1-qu3)	P-value for SNP	iSNV count median (qu1-qu3)	P-value for iSNVs
Total	102(100)				
Age groups (years)					
<18	12 (11.8)	4.5 (3-6.75)	0.4808	2 (1-4)	0.3165
18-40	44 (43.1)	5.5 (3-8)	ref	1 (0-4)	ref
41-65	31 (30.4)	4 (2.5-6)	0.2232	2 (1-3.5)	0.5971
>65	15 (14.7)	4 (2-6.5)	0.2084	2 (0.5-3.5)	0.7816
Sex					
Male	52 (51.0)	4 (3-7)	ref	1 (0-4)	ref
Female	50 (49.0)	5 (3-7)	0.9196	2 (1-4)	0.5564
Disease level					
Mild	33 (32.4)	6 (3-8)	0.0068	2 (0-4)	0.9333
Moderate	51 (50)	4 (2.5-5.5)	ref	2 (1-4)	ref
Severe	8 (7.8)	5.5 (3-7)	0.5469	2.5 (1-3.5)	0.6365
Critical	10 (9.8)	4 (3.25-5.5)	0.7014	1 (0-2.75)	0.1945
Classification					
Wuhan	20 (19.6)	3 (1-4)	0.0832	1 (0.5-3.5)	0.1562
Local	43 (42.2)	4 (2.5-5)	ref	3 (1-4.25)	ref
Import	39 (38.2)	7 (5-8)	4.32E-05	2 (0.5-4)	0.6396

Supplementary Table 5. Primers for PCR validation of three large long deletions.

Deletion size (nt)	Primer ID	Primer sequence
28	D1F	CAGGTTTTGCTGCATACAGTC
	D1R	GCTCACAAGTAGCGAGTG
120	D2F	CTCGCTACTTGTGAGCTTTATC
	D2R	GGTTGATGTTGAGTACATGACTG
154	D3F	GAAACTTGTCACGCCTAAAC
	D3R	GGTGCCAATGTGATCTTTTG
