# Supplementary material for: Genomic surveillance of COVID-19 cases in Beijing
Source: Nat Commun. 2020 Oct 30;11:5503. doi: 10.1038/s41467-020-19345-0 (PMC7603498; doi:10.1038/s41467-020-19345-0)
Supplement: Supplementary file 2 — Descriptions of Additional Supplementary Files [file 41467_2020_19345_MOESM2_ESM.pdf]

Descriptions of Additional Supplementary Files

Supplementary data 1
Description: SNPs identified in this study.

Supplementary data 2
Description: The background information of publicly SARS-CoV-2 genomes from GISAID used in this study.

Supplementary data 3
Description: iSNVs identified in this study.

Supplementary data 4
Description: indels identified in this study.
