# Supplementary material for: Genomic surveillance of COVID-19 cases in Beijing
Source: Nat Commun. 2020 Oct 30;11:5503. doi: 10.1038/s41467-020-19345-0 (PMC7603498; doi:10.1038/s41467-020-19345-0)
Supplement: Supplementary file 8 — Reporting Summary [file 41467_2020_19345_MOESM8_ESM.pdf]

nature research

Corresponding author(s): Chen Chen, Hui Zeng, Di Liu, Jianbin Wang

Last updated by author(s): 2020/09/29

Reporting Summary

Nature Research wishes to improve the reproducibility of the work that we publish. This form provides structure for consistency and transparency in reporting. For further information on Nature Research policies, see our Editorial Policies and the Editorial Policy Checklist.

Statistics

For all statistical analyses, confirm that the following items are present in the figure legend, table legend, main text, or Methods section.

n/a	Confirmed	
☐	☒	The exact sample size (n) for each experimental group/condition, given as a discrete number and unit of measurement
☐	☒	A statement on whether measurements were taken from distinct samples or whether the same sample was measured repeatedly
☐	☒	The statistical test(s) used AND whether they are one- or two-sided<br>*Only common tests should be described solely by name; describe more complex techniques in the Methods section.*
☒	☐	A description of all covariates tested
☐	☒	A description of any assumptions or corrections, such as tests of normality and adjustment for multiple comparisons
☐	☒	A full description of the statistical parameters including central tendency (e.g. means) or other basic estimates (e.g. regression coefficient) AND variation (e.g. standard deviation) or associated estimates of uncertainty (e.g. confidence intervals)
☐	☒	For null hypothesis testing, the test statistic (e.g. F, t, r) with confidence intervals, effect sizes, degrees of freedom and P value noted<br>*Give P values as exact values whenever suitable.*
☒	☐	For Bayesian analysis, information on the choice of priors and Markov chain Monte Carlo settings
☒	☐	For hierarchical and complex designs, identification of the appropriate level for tests and full reporting of outcomes
☒	☐	Estimates of effect sizes (e.g. Cohen's d, Pearson's r), indicating how they were calculated

Our web collection on statistics for biologists contains articles on many of the points above.

Software and code

Policy information about availability of computer code

Data collection: Clinical data were collected from the electronic medical records system of our hospital, and no commercial or custom code was used.

Data analysis: We used the following software to analyze the sequencing data which have been described in Method, including Bowtie 2 v2.1.0 and Samtools v1.3.1 for read mapping, the iSNV calling approach (https://github.com/generality/iSNV-calling), BLAST v2.2.18 and MView v1.67 for sequence comparison and calling of SNPs and iSNVs, BCFtools v0.1.19 for generating the consensus sequences, MAFFT v7.453 for multiple sequence alignments, IQ-TREE v1.6.12 for phylogenetic analysis, and pangolin v2.0.4 for lineage determination. In house perl script (perl v5.26.2) was used for automatically batch analysis using above software. R package stats v3.5.3 was used for statistical analysis.

For manuscripts utilizing custom algorithms or software that are central to the research but not yet described in published literature, software must be made available to editors and reviewers. We strongly encourage code deposition in a community repository (e.g. GitHub). See the Nature Research guidelines for submitting code & software for further information.

Data

Policy information about availability of data

All manuscripts must include a data availability statement. This statement should provide the following information, where applicable:

- Accession codes, unique identifiers, or web links for publicly available datasets
- A list of figures that have associated raw data
- A description of any restrictions on data availability

The viral sequencing data we obtained in this study has been deposited in GenBank database under the accession number PRJNA667180 (https://www.ncbi.nlm.nih.gov/bioproject/PRJNA667180). The published SARS-CoV-2 genomes used in this study were downloaded from GISAID (https://www.gisaid.org/) with suitable acknowledgments (Supplementary Data).
Fiugre 1B, 1C, 1D, 2, 3B, 3C and S3-S6 have associated raw data in the Source Data file.

Field-specific reporting

Please select the one below that is the best fit for your research. If you are not sure, read the appropriate sections before making your selection.

☒ Life sciences ☐ Behavioural & social sciences ☐ Ecological, evolutionary & environmental sciences

For a reference copy of the document with all sections, see nature.com/documents/nr-reporting-summary-flat.pdf

Life sciences study design

All studies must disclose on these points even when the disclosure is negative.

Sample size	We enrolled 178 COVID-19 cases.
Data exclusions	The genomic sequences from GISAID were filtered by the sequence quality: (1) completely assembled, (2) anonymous nucleotides ≤ 15, (3) degenerate bases ≤ 50, (4) gaps (including deletion and insertion) ≤ 2, (5) coverage of mutation regions < 25%, (6) sequence length ≥ 25,000 bp. This criteria was referred to that of the National Genomics Data Center (https://bigd.big.ac.cn/). The data we obtained by meta-transcriptomic sequencing were filtered by sequencing depth >=5 and reference coverage >=85%. This is a empirical criteria to exclude low quality data by random error.
Replication	The data and the methods we used in this study are all available and fully described to verify the reproducibility. We have repeated the analysis three times and confirmed the results. The meta-transcriptomic sequencing was not performed repeatedly due to the high costs. The quality control was conducted by adding negative control of water in each run and several pharyngeal swabs from the healthy in random runs.
Randomization	This is not relevant to our study. This study focused on the epidemiological investigation of COVID-19 cases and the viral genomes, and no experimental groups were required.
Blinding	This is not relevant to our study. This study is a observational study and the cases were enrolled based on the explicit diagnosis of COVID-19. The clinical situations were informed to the patients during the treatments, and interviews were conducted for epidemiological histories. Therefore, the blinding was not required and not applicable in this study.

Reporting for specific materials, systems and methods

We require information from authors about some types of materials, experimental systems and methods used in many studies. Here, indicate whether each material, system or method listed is relevant to your study. If you are not sure if a list item applies to your research, read the appropriate section before selecting a response.

Materials & experimental systems

n/a	Involved in the study	
☒	☐	Antibodies
☒	☐	Eukaryotic cell lines
☒	☐	Palaeontology and archaeology
☒	☐	Animals and other organisms
☐	☒	Human research participants
☒	☐	Clinical data
☒	☐	Dual use research of concern

Methods

n/a	Involved in the study	
☒	☐	ChIP-seq
☒	☐	Flow cytometry
☒	☐	MRI-based neuroimaging

Human research participants

Policy information about studies involving human research participants

Population characteristics	All of the patients enrolled in this study were confirmed COVID-19 cases, including 52 males and 50 females. The mean age of the patients is 40.0±22.0 years (range: 0.8-88).
Recruitment	We enrolled all of the 178 COVID-19 cases with available samples in our hospital from January 29 to April 17, 2020.
Ethics oversight	This study was approved by the Ethics Committee of Beijing Ditan Hospital, Capital Medical University.

Note that full information on the approval of the study protocol must also be provided in the manuscript.
